# Nasopharyngeal Pneumococcal Carriage in Nigeria: a two-site, population-based survey

**DOI:** 10.1038/s41598-018-21837-5

**Published:** 2018-02-22

**Authors:** Ifedayo M. O. Adetifa, Aishatu L. Adamu, Angela Karani, Michael Waithaka, Kofo A. Odeyemi, Christy A. N. Okoromah, Mohammed M. Bello, Isa S. Abubakar, Victor Inem, J. Anthony. G. Scott

**Affiliations:** 1 Department of Infectious Diseases Epidemiology, London School of Hygiene and Tropical Medicine, WC1E 7HT, London, UK; 2 Department of Epidemiology and Demography, KEMRI-Wellcome Trust Research Programme, Po Box 230-80108, Kilifi, Kenya; 3 Department of Paediatrics and Child Health, College of Medicine University of Lagos/Lagos University Teaching Hospital, Idi-Araba, PMB 12003 Lagos, Nigeria; 4 Department of Community Medicine, Bayero University/Aminu Kano Teaching Hospital, PMB 3452 Kano, Nigeria; 5 Department of Community Medicine and Primary Care, College of Medicine University of Lagos/Lagos University Teaching Hospital, Idi-Araba, PMB 12003 Lagos, Nigeria

## Abstract

Changes in nasopharyngeal (NP) carriage of vaccine-type (VT) *Streptococcus pneumoniae* can be used to assess the effectiveness of a pneumococcal conjugate vaccine (PCV10). We conducted a baseline carriage survey in rural (Kumbotso, Kano) and urban (Pakoto, Ogun) Nigeria. In this cross-sectional study, we obtained data on demography, clinical history, risk factors, and took NP swabs for pneumococcal culture. We calculated crude and age-standardised carriage prevalence and used log-binomial regression to assess risk factors for carriage. Among children aged <5 years, 92% (95% CI: 88–95%) and 78% (73–82%), respectively, carried any pneumococcus and 48% and 50%, respectively, carried PCV10 serotypes. In Kumbotso, carriage prevalence was >40% across all ages. The age-standardized prevalence of pneumococcal carriage was 66% in Kumbotso and 40% in Pakoto. The most commonly identified serotypes were 19 F, 6 A and 23 F. Risk factors for carriage were young age, recent rhinorrhoea, cohabitation with ≥2 children aged <5 years, and sharing a bed with ≥2 persons. Pneumococcal carriage prevalence is high in this Nigerian population. Persisting prevalence of VT-carriage in older children and adults suggests that PCV10 introduction in children will not eliminate transmission of vaccine serotypes rapidly. High vaccine coverage will therefore be required to ensure full protection of children.

## Introduction

Pneumococcal conjugate vaccines (PCVs), first introduced in high income countries, have substantially reduced the burden of invasive pneumococcal diseases (IPD) and pneumonia^1^. PCV introductions in Africa have had varied but significant direct and indirect effects on IPD^2,3^ and pneumonia^4^ caused by vaccine type (VT) serotypes. PCVs have reduced the carriage prevalence of VT serotypes in both the vaccinated and unvaccinated^5,6^.

With ~6.7 million cases causing >100,000 deaths per annum, Nigeria has the highest estimated burden of childhood pneumococcal diseases in Africa^7,8^. So, Nigeria commenced a phased introduction of a 10-valent PCV (PCV10) in 2015 that attained nationwide spread in 2016. Gavi, The Vaccine Alliance made this possible by supplying PCV to Nigeria and other eligible countries at less than a tenth of the purchase price^9^. However, Nigeria like many other African countries, has not invested in the longitudinal surveillance systems required to determine effectiveness of the PCV immunisation programme and provide an evidence base to sustain this investment^2,3,6^.

Although Gavi has supported introductions of new vaccines, there is an associated risk to sustainability of programmes with expensive new vaccines like PCV. For example, with Nigeria in the accelerated phase of transition from Gavi support, current subsidy will drop from $199 million in 2017 to $74 million in 2021. Concurrently, the country’s commitment to vaccine purchase will rise >2-fold from $123 million $279 million from 2017 to 2021. Therefore, the need to generate context-specific vaccine effectiveness and cost effectiveness data to support the case for continued investment in these vaccines at the end of this transition cannot be over emphasised. Unfortunately, establishing IPD surveillance is not just late but too expensive and impracticable at this point.

Carriage is on the causal pathway to disease, and is also a reflection of transmission^10^. So, changes in VT-carriage prevalence can be used to examine the impact of vaccine on transmission and to estimate the likely changes in disease risk, through modelling. The modelling methods developed to predict PCV10 effectiveness against disease by studying changes to NP carriage of VT-pneumococci vary and are attractive alternatives to IPD data because they do not require expensive surveillance systems. They utilise a mixture of pre-vaccine serotype-specific IPD incidence and carriage, population mixing patterns, serotype specific case-to-carrier ratios, post-vaccine carriage data, etc., to determine vaccine effectiveness^11–16^. Although an emerging area of research especially in low-income settings with high disease burden, we and others have validated some of these modelling approaches using observed carriage and IPD data^11,16^ and there is merit in expanding their application in these settings.

As part of a plan to assess PCV10 impact using serial pneumococci carriage data, we conducted baseline cross-sectional surveys of NP carriage in two sites-one each in northern and south-western Nigeria. We had previously studied the south-western site (Pakoto) so it will provide a more stable estimate of baseline prevalence.

## Methods

We studied two centres in Nigeria, one urban (Pakoto, Ogun State) and one rural (Kumbotso, Kano State). The methods followed those of a previous study conducted in Pakoto in March 2009^17^. We defined the study areas based on a single reference point: for Pakoto this was the Lagos University Teaching Hospital (LUTH) Primary Health Care and Rural Medicine; for Kumbotso it was the Aminu Kano Teaching Hospital (AKTH) Comprehensive Primary Health Care Centre. All residents living within 10 km of these reference points were eligible for inclusion in the study. The LUTH centre serves communities spread across Ifo and Ado-Odo/Ota Local Government Areas (LGA) ~40 km away from Lagos, with population densities of 1007/ km^2^ and 600/km^2^, respectively. AKTH serves Kumbotso LGA ~22 km from Kano, with a population density of 1873/km^2^.

A sampling frame was not available for both study sites; we therefore sampled the population based on voluntary participation. Following community consultation, sensitisation of community leaders and announcements by town criers, the study team set-up a research base on pre-agreed dates at pre-selected locations. We then sampled all eligible members of the community who came to the study site until either the target sample size was achieved or the pre-determined period for field activities came to an end. We selected a sample size of 1000 per site to estimate carriage prevalence with adequate precision for the least prevalent VT serotype (0.38% based on results from earlier survey^17^): for example, the 95% Confidence Intervals (CI) for an estimate of 0.4% prevalence would be 0.1–1.0)

The study was conducted at Kumbotso in December 2016 and at Pakoto in February 2017. These selected study sites were part of the last phase of PCV10 (Synflorix®) introduction. Kumbotso began PCV10 vaccinations in July 2016 and Pakoto in October 2016. WHO/ UNICEF and administrative reports put national coverage of the 3^rd^ dose of PCV10 at 13% and 73% respectively in 2015^18^. At the time of sampling, infants had only recently become eligible for vaccination because Kumbotso and Pakoto had experienced just 5 and 4 months respectively of PCV10 introduction. Only 6.5% (41 of 630) of children <5 years had vaccine records and only 12% (5 of 41) had received 2 or more doses of PCV10.

Questionnaires administered in a language understood by all consenting participants were used to obtain data on sex, age, household size and composition; education, occupation, history of oral antibiotic treatment and respiratory symptoms in the two weeks preceding the study.

Following World Health Organisation guidelines, we obtained nasopharyngeal specimens from all consenting study participants using nylon-tipped flexible nasopharyngeal flocked swabs (Copan flock technologies, Cat. No. 503CS01, Brescia, Italy)^19^. Following sampling, nasopharyngeal swab tips were immediately immersed in skim milk tryptone glucose glycerol (STGG) transport media. In Kumbotso, samples were transported within 8 hours of collection respectively to the AKTH Microbiology Laboratory, Kano while for Pakoto, they were transported to and stored at the Covenant University Health Centre laboratories, Sango Ota till the end of fieldwork following which they were moved to the Central Research Laboratory, College of Medicine University of Lagos, Lagos. In these locations, the STGG samples were frozen at −20^0^C till they were shipped on dry ice to the microbiology laboratory at KEMRI-Wellcome Trust Research Programme (KWTRP), Kilifi, Kenya for processing^19^. Samples from Kumbotso were stored in country for 8 weeks and those from Pakoto for 6.5 weeks.

*S. pneumoniae* isolates were identified from gentamicin-blood agar by optochin susceptibility testing; serotyping was done by latex agglutination and the Quellung reaction (including specific antisera for all members of serogroup 6). Where colonies varying in appearance were seen, the dominant morphotype was serotyped. In case of optochin intermediate susceptibility, bile solubility was used for confirmation. Where Quellung reaction was inconclusive, suspected pneumococci were tested by polymerase chain reaction (PCR) targeting the genes encoding autolysin (lytA). If lytA-positive, the isolates were then serotyped using multiplex PCR^20^.

Statistical analyses were performed using Stata 13.1 (Stata Corp LP, College Station, Texas). The main outcome variable was overall, vaccine type and location-specific nasopharyngeal carriage prevalence and associated 95% confidence intervals (95% CI). Vaccine serotypes were defined for the following vaccines; Synflorix® PCV10 (1, 4, 5, 6 B, 7 F, 9 V, 14, 18 C, 19 F, and 23 F), Prevnar 13® PCV13 (PCV10 + 3, 6 A, 19 A) and a PCV10 (SII), a variant by Serum Institute India (1, 5, 6 A, 6 B, 7 F, 9 V, 14, 19 A, 19 F and 23 F) currently in clinical development. Carriage prevalence was also estimated across the following age-strata: 0–4, 5–17, 18–34, 35–49 and ≥50 years. To adjust for differing age structure by study location, carriage prevalence were age-standardised using the age strata for INDEPTH network’s standard population for low- and middle income countries as weights^21^.

We examined associations between risk factors and carriage by estimating relative risk. Risk factors examined were age, sex, household smoking, household cooking fuel, number of under-five year-olds in the household, number of persons sharing a bed, history of upper respiratory tract infection and antibiotic use in the preceding 2 weeks. We tested the association between the distribution of participant characteristics of interest and the study locations with chi squared tests. Adjusted prevalence ratios were calculated with log-binomial regression models. Where these failed to converge, Poisson regression models with robust 95%CIs were used instead. Prevalence ratios were adjusted for all potential confounding variables that were significant at a p-value <0.05.

The study was approved by the following: London School of Hygiene and Tropical Medicine Ethics Committee, Kenya Medical Research Institute’s Scientific and Ethical Review Unit, Research and Ethics Committees of Aminu Kano and Lagos University Teaching Hospitals, and by the Kano State Ministry of Health. All study procedures including laboratory methods were performed in accordance with the relevant guidelines and regulations. Parents/guardians of minors and every adult participant gave written informed consent before participation in study. Participants aged 15–17 years gave written assent in addition to the written consent given by parents/guardians.

### Data availability statement

This is an ongoing study so data is not publicly available. However, data can be made available from the KWTRP Institutional Data Governance Committee to researchers who meet the criteria for access to data. Interested researchers can contact, Ms. Elizabeth Mwatata (EMwatata@kemri-wellcome.org).

## Results

### General characteristics of the study population

The epidemiological characteristics of participant overall and by location are shown in Table 1. There were more females, household crowding, more upper respiratory symptoms and greater reliance on firewood for cooking in the rural population (Kumbotso) and greater access to antibiotics and flush toilets in the urban population (Pakoto).

**Table 1:** General Characteristics of the Nigerian pneumococcal carriage survey population.

Characteristics*	Location				p-value for difference**
	Kumbotso (rural)		Pakoto (urban)		
	N	%	N	%	
**Sample size**	876	*48.7*	924	*51.3*	
**Sex**					<0.0005
Female	548	*62.6*	521	*56.4*	
**Age group** (**years**)					<0.0005
0–4	295	*33.9*	335	*36.5*	
5–17	288	*33.1*	250	*27.2*	
18–34	145	*16.7*	109	*11.9*	
35–49	76	*8.7*	84	*9.1*	
≥50	66	*7.6*	141	*15.3*	
**Clinical history (in preceding 2 weeks**)					
Cough	448	*51.1*	216	*23.4*	<0.0005
Rhinorrhoea	712	*81.3*	238	*25.8*	<0.0005
Antibiotic use	65	*7.4*	145	*15.7*	<0.0005
**Main cooking fuel**					<0.0005
Firewood	828	*94.5*	40	*4.3*	
Kerosene	16	*1.8*	515	*55.7*	
Cooking gas	12	*1.4*	326	*35.3*	
**Household amenities**					
Main source of drinking water					NS
Piped water	789	*90.1*	818	*88.5*	
Dug well	74	*8.5*	73	*7.9*	
Main means of sewage disposal					<0.0005
Flush/pour flush toilet	89	*10.2*	777	*84.1*	
Pit latrine	756	*86.3*	140	*15.2*	
**Household composition**					
Have ≥2 children <5 years in household	643	*73.4*	95	*10.3*	<0.0005
Have ≥2 sharing a bed	727	*83.0*	185	*20.1*	<0.0005
**Tobacco exposure**					
Smoker (if aged ≥12 years)	9	*2.6*	5	*1.3*	NS
Smoker in the household	65	*7.4*	20	*2.2*	<0.0005

*Subtotals for characteristics may not add up to N on account of missing data.

**p-values are for differences in group-level characteristics between locations unless individual level values are provided.

### Carriage Prevalence

Out of 1800 participants, 1109 [61.6% (95% CI: 59.3–63.9)] were found to be carrying pneumococci. The crude carriage prevalence in Kumbotso, 73.7% (95% CI: 70.7–76.6) was higher than seen in Pakoto, 50.1% (95% CI: 46.8–53.4). As shown in Table 2, crude prevalence was highest for the youngest age groups in each site and was higher at all ages in Kumbotso than in Pakoto. Although carriage declined sharply at both sites from approximately 18 years onwards, the persistence of high carriage prevalence at older age groups was particularly noticeable in Kumbotso. We observed a marked association between carriage and sex in Pakoto (58.2% (95% CI: 53.3–63.2) in males compared to 43.9% (95% CI: 39.7–48.2) in females) but little difference in Kumbotso (males 76.7% compared to females 72.1%). Similar to the crude estimates, age-standardised carriage prevalence in Kumbotso was higher than in Pakoto (65.7% [95% CI: 55.9–75.2] vs. 40.2% [95% CI: 30.3–50.3]).

**Table 2:** Crude carriage prevalence in study locations by age and sex.

	Kumbotso (rural)				Pakoto (urban)			
	Male		Female		Male		Female	
	N	%	N	%	N	%	N	%
Age group*								
All ages	250	*76.7*	395	*72.1*	219	*58.2*	229	*44.0*
0–4 years	131	*91.6*	139	*92.1*	132	*77.2*	117	*78.0*
5–17 years	95	*75.4*	133	*82.1*	70	*53.4*	51	*46.4*
18–34 years	11	*39.3*	66	*56.4*	2	*25.0*	25	*25.0*
35–49 years	4	*57.1*	30	*43.5*	5	*31.3*	14	*20.6*
≥50 years	9	*40.9*	24	*54.6*	9	*19.2*	21	*23.1*

*Subgroup subtotals may not add up to ‘Ns’ because of missing data (3 females for Kumbotso and 1 each for males and females in Pakoto have missing age data).

### Pneumococci Serotypes in carriage and proportions included in PCVs

**Figure 1:**
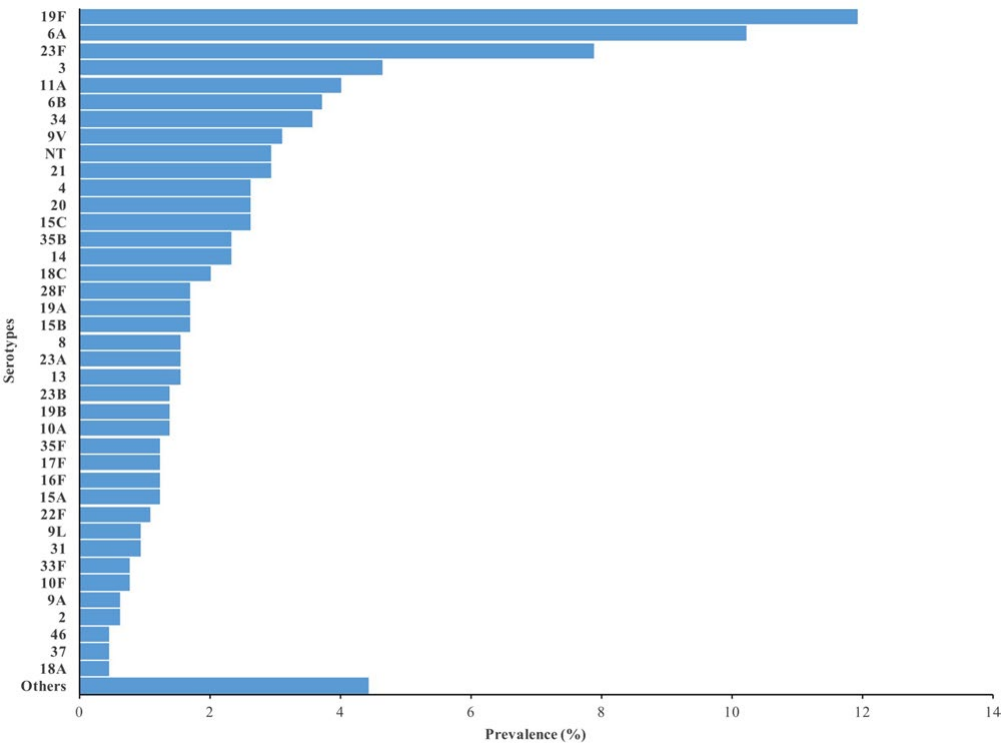
Nasopharyngeal carriage prevalence of individual Pneumococcal serotypes in Kumbotso (rural setting), Nigeria.

**Figure 2:**
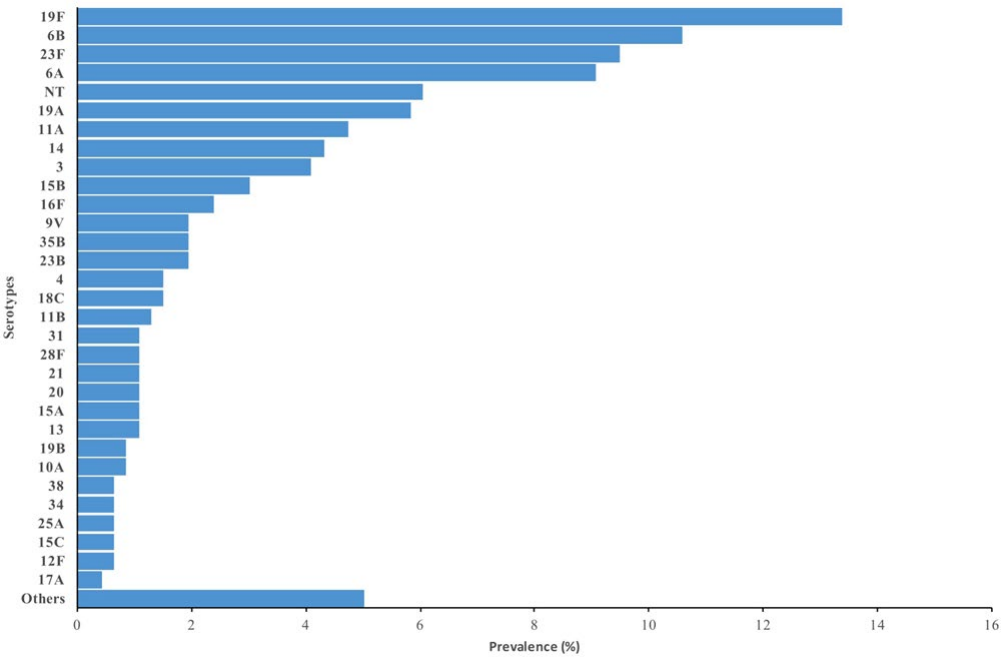
Nasopharyngeal carriage prevalence of individual Pneumococcal serotypes in Pakoto (urban setting), Nigeria.

The 1109 pneumococci isolates found represented 65 different serotypes and 47 (4.0%) of the isolates were non-typeable (NT). Fifty-eight and 48 different serotypes were seen in Kumbotso and Pakoto, respectively, and the prevalence of each serotype in rank order by location is shown in Figs 1 and 2. The collection of isolates was dominated by vaccine serotypes. No serotype 6 C was found and there were only 2 (0.3%) and 1 (0.2%) isolates of serotypes 1 and 5, respectively. There were some differences in serotype-specific prevalence by age and study location but the patterns observed for children aged <5 years in both locations were similar (see Table S1).

Carriage prevalence of serotypes contained in PCVs by location and age group can be seen in Table 3. The proportion of PCV10 (Synflorix®) serotypes carried by participants was slightly higher in Pakoto than Kumbotso for all age categories. In addition, a higher proportion of participants carried serotypes found in PCV10 (SII) compared to Synflorix®. Regardless of PCV formulation, carriage prevalence of vaccine serotypes was highest the youngest age categories.

**Table 3:** Carriage Prevalence of Vaccine-Type Serotypes by location and by age-group.

Location	Kumbotso (rural)				Pakoto (urban)			
Vaccine preparation	N	PCV10	PCV10 (SII)	PCV13	N	PCV10	PCV10 (SII)	PCV13
		%	%	%		%	%	%
**Age group**								
0–4 years	271	48	61	68	260	50	65	69
5–17 years	228	31	35	48	123	41	50	58
18–34 years	77	26	25	35	27	33	37	44
35–49 years	34	27	27	35	19	32	32	42
≥50 years	33	6	12	21	32	25	28	56

### Assessment of risk factors for carriage

In univariate analyses, age <35 years, being male, residence in a rural study site (Kumbotso), a recent history of a cough and runny nose, living in households with 1 or more children under-fives, sharing a bed with ≥2 persons and the use of firewood as the main cooking fuel, were all associated with carriage (Table 4). In multivariate analyses, we tested for interactions between sex and location because of the earlier described association between gender, settlement and carriage. The following were associated with carriage: age <18 years, residence in Kumbotso, recent history of a runny nose, living in a household with ≥2 children aged under 5 years, and sharing a bed with ≥2 persons in a household (Table 4). Of all of the age groups below 18 years, carriage prevalence in the 0–4-year-olds was the highest (2.3 times that in oldest age group). There was no interaction between sex and study location (RR 0.83 [95%CI 0.64–1.01], p = 0.13 for Likelihood ratio test).

**Table 4:** Risk factors for pneumococcal carriage across all ages in Nigerian populations.

Characteristic	n/N*	%	Pneumococcal carriage- Prevalence Ratios (95% CI)			
			Univariate analysis		Multivariable analysis^†^	
			Overall	p-value	Overall	p-value
**Age group (years)**						
≥50	65/207	*31.4*	1.00		1.00	
35–49	53/160	*33.1*	1.06 (0.78–1.42)	0.726	0.96 (0.71–1.30)	0.788
18–34	104/254	*40.9*	1.30 (1.02–1.67)	**0.037**	1.14 (0.88–1.47)	0.324
5–17	351/538	*65.2*	2.08 (1.68–2.57)	**<0.0005**	1.79 (1.44–2.23)	**<0.0005**
0–4	532/630	*84.3*	2.68 (2.19–3.29)	**<0.0005**	2.26 (1.82–2.81)	**<0.0005**
**Settlement**						
Pakoto	463/924	*50.1*	1.00		1.00	
Kumbotso	646/876	*73.7*	1.50 (1.36–1.58)	**<0.0005**	1.40 (1.11–1.65)	**<0.0005**
**Sex**						
Male	469/702	*66.8*	1.00		1.00	
Female	624/1069	*58.4*	0.87 (0.81–0.94)	**<0.0005**	1.02 (0.95–1.09)	0.58
**Runny nose in the preceding 2 weeks**						
No	416/838	*49.6*	1.00		1.00	
Yes	687/950	*72.3*	1.46 (1.35–1.58)	**<0.0005**	1.13 (1.02–1.25)	**0.018**
**Cough in the preceding 2 weeks**						
No	622/1125	*55.3*	1.00		1.00	
Yes	482/664	*72.6*	1.31 (1.2–1.41)	**<0.0005**	0.98 (0.91–1.06)	0.656
**Antibiotics use in the preceding 2 weeks**						
No	964/1567	*61.5*	1.00			
Yes	135/210	*64.3*	1.05 (0.94–1.16)	0.425		
**Number of<5 years residents in household**						
0	282/607	*46.5*	1.00		1.00	
1	264/428	*61.7*	1.33 (1.19–1.49)	**<0.0005**	1.09 (0.97–1.22)	0.157
≥2	552/738	*74.8*	1.61 (1.46–1.77)	**<0.0005**	1.15 (1.02–1.30)	**0.028**
**Number of persons sharing a bed**						
0–1	425/863	*49.3*	1.00		1.00	
2	487/662	*73.6*	1.49 (1.38–1.62)	**<0.0005**	1.12 (1.01–1.24)	**0.034**
≥3	191/250	*76.4*	1.55 (1.41–1.71)	**<0.0005**	1.15 (1.02–1.29)	**0.019**
**Exposure to cigarette smoking**						
No	1047/1703	*61.5*	1.00			
Yes	56/85	*65.9*	1.07 (0.92–1.25)	0.390		
**Main household cooking fuel**						
Gas	160/338	*47.3*	1.00		1.00	
Kerosene	283/531	*53.3*	1.13 (0.98–1.29)	0.092	1.09 (0.96–1.24)	0.201
Firewood	627/868	*72.2*	1.53 (1.35–1.72)	**<0.0001**	0.90 (0.74–1.10)	0.315
Others (Charcoal, sawdust, crop)	15/24	*62.5*	1.32 (0.95–1.84)	0.137	1.26 (0.96–1.65)	0.093

^†^Results of a Poisson model with robust 95% CI are presented.

*N may differ from total sample size 1800 because of missing data.

## Discussion

Given the estimated burden of pneumococcal diseases in Nigeria^7,8^, there is a dearth of clinical surveillance data in the country. This dearth of data extends to local carriage data^22–24^.

This study was conducted in anticipation of using changes in NP carriage of pneumococci observed in serial surveys to assess the effectiveness of the PCV10 immunisation programme. Having anticipated the introduction of a PCV and knowing it was impractical and expensive to commence IPD surveillance, we had earlier acquired pre-vaccine carriage data and described the population biology of carried pneumococci in southwestern Nigeria^17^. We now report on a second baseline survey with expanded scope to a second study site based in northern Nigeria and anticipate producing estimates of impact on disease once we have a significant ‘post-vaccine’ survey.

We found crude pneumococcal carriage prevalence in both rural and urban study locations and across all age groups was high. The very high carriage prevalence in children aged <5 years in Kumbotso (rural) is consistent with findings in similar settings in Gambia^25–27^ and Ethiopia^28,29^ and most likely reflects the intensity of transmission/the force of infection in these settings. However, it higher than that seen in rural Kenya^6,30^ and in ethnic minorities resident in similarly deprived/underserved areas^31,32^.

For comparability between populations with different population structures, we calculated age-standardised carriage prevalence. The age-standardised carriage was much higher in our rural northern Nigeria location than in a rural Kenyan setting^6^, than in combined studies from West Africa and the rest of sub-Saharan Africa^33^. They also exceed the 44.4% reported by Hansman in Ibadan, South West Nigeria in 1977^22^, and the 69% recorded recently in infants in south eastern Nigeria^23^. The age-standardised carriage prevalence here also validates the age-standardised results of our earlier survey conducted in the same season, and urban location-Pakoto (40.2% now vs. 37.0 previously). In addition, crude PCV10 serotype coverage also remained stable between both surveys in this location (43.8% vs. 44.5% respectively)^17^.

Almost half of study population aged ≥ 5 years carried pneumococci which is significant especially as the crude carriage prevalence for the 5–17-year-olds, a third of our study population, ranged from 49.2–79.2%. Social contact data in similar settings of high transmission show children <5 years have the highest daily contacts across all ages^34^ and that household transmission of pneumococci is mostly from older to younger children^35^. Taken together with our results, these suggest the older age groups, particularly the 5–17-year-olds, may serve as reservoirs that will facilitate transmission of VT-pneumococci to susceptible under-fives as seen even in the vaccine era^6^. Therefore, with higher carriage prevalence in children <5 years, the well-known challenges of attaining high vaccination coverage in Nigeria and the fact that vaccine effects may take 3–4 years to become apparent at high coverage^2^, it is unlikely the PCV10 progammme can achieve near elimination of VT pneumococci in carriage as seen elsewhere^36^. Crude carriage in adults, 35.8% (range 23.4 −50.2%) is similar to reports from Nigeria (15–25-year- olds)^24^, Gambia^25^ but higher than observed in the rest of sub-Saharan Africa^33^.

The differences in the study setting for each of the Nigerian studies is most likely an important contributor to the difference in results. Furthermore, our study was conducted during the Harmattan season. This cold season runs from the end of November into the middle of March and gets its name from a dry and dusty northeasterly trade wind that blows from the Sahara Desert in a south-westward direction to the Gulf of Guinea^37,38^. Kumbotso within Nigeria’s Sahel Savannah endures a greater share of this weather front compared to Pakoto or any other location further south. This season is also associated with increased pharyngeal carriage of *Neisseria meningitidis* and meningitis outbreaks in the meningitis belt of Africa within which Kumbotso in Kano State falls^39,40^. Some of the risk factors that were strongly associated with carriage were also rarely found in the urban areas and this may also explain the higher prevalence of carriage in the rural area.

In this one study, we found 65 pneumococcal serotypes similar to the 70 identified in review of studies in sub-Saharan Africa^33^. This expands our understanding of the diversity of prevalent pneumococci in carriage in Nigeria. Despite expanded testing for serogroup 6 serotypes, we did not find any pneumococci of serotype C. This is consistent with our earlier survey. Serotypes 1 and 5 are commonly isolated in disease in Africa^41–44^ where they cause about 13% and 10% of meningitis and other IPD respectively^45^. However, we found carriage of these serotypes was very low in our study population similar to other reports^17,25,30,44^.

Unfortunately, data on serotypes responsible for IPD in Nigeria^46–48^ are also very limited. Pooled data from Africa (>90% southern Africa studies) show the top ranked serotypes responsible for childhood IPD are 14, 6B, 6 A, 23 F, 19 F and 19 A^45^. PCV10 covers serotypes responsible for 62% and 67% of meningitis and other IPD respectively, while it is 72% and 81% for these conditions respectively for PCV13^45^. That the proportion of serotype covered is this high for IPD compared to carriage is not surprising as both vaccines significantly reduce the incidence of VT-IPD^2,3,11,49^, pneumonia^4^ and VT-carriage^6,50^ in vaccinated and unvaccinated children. Estimating the full extent PCV impact on IPD with carriage data is challenging because relative to IPD, VT-serotypes are less represented in carriage especially serotypes 1 and 5^19,51^. Therefore, some caution will be required if unadjusted carriage data is used for assessments of impact of introducing PCVs introduction and in particular for PCVs serotype-specific comparisons. However, studies such as ours are essential to provide information about serotype changes and potential impact on changing epidemiology in a relatively inexpensive manner, and to inform future vaccine development.

In theory, there would be a greater impact for PCV(SII) and PCV13 against IPD compared to Synflorix® based on carriage data but there are also other considerations. For example, an expanded impact on IPD mediated by induction of cross protection against vaccine-related serotypes- 6 A and 19 A has been attributed to Synflorix® ^52,53^. However, our carriage data in Kenya^6^ suggests otherwise. In addition, PCV13 despite containing serotype 3 is thought to confer very little or no protection against this serotype^54^. In contrast, surveillance data from The Gambia shows a decline and subsequent elimination of serotype 3 IPD following PCV13 introduction^3^. Despite the foregoing, the overall impact of vaccination against IPD is similar irrespective of PCV-type. This underpins the current consensus to prioritise PCV introductions in all settings and especially in those with the highest under-5 mortality, and to ensure high vaccination coverage^1,54,55^.

The main risk factors for carriage in this survey were young age (<18 years), history of rhinorrhoea, rural residence, living in household with ≥2 children aged <5years and sharing a bed with ≥2 persons. These are all well described risk factors for carriage^17,31^.

Obtaining a randomly selected population sample in Nigeria is a major challenge in research and this explains why only a few have succeeded in doing this in the past. That our sampling of participants was not probability-based is the principal limitation of this study. Although young children are the usual focus of carriage studies, another limitation was the small numbers of adult male participants compared to their female counterparts. Considering our participants were volunteers (not induced by payments) responding to communication via community leaders and town criers, and were healthy and/or ambulatory, any selection bias is likely to lead to an underestimate of carriage prevalence. Since the extent to which biases present in these study locations may change overtime is unknown, we will explore the use of satellite photos and other options to create suitable sampling frames that will allow for random sampling in follow-up surveys.

## Conclusions

Pneumococcal carriage is highly prevalent in the age group targeted for vaccination. The high carriage prevalence seen in the older age groups suggests the probability of eliminating carriage of VT serotypes in the population by vaccinating birth cohorts alone (as happened in the USA) is very low. Therefore, the indirect benefits of vaccine are likely to be limited – and the benefits of the vaccine programme will be in proportion to the vaccine uptake, which is quite poor in Nigeria. Priority should be given to ensuring high vaccination coverage in this setting. These findings highlight the importance of these carriage data - and their future complements - to test and inform policy in PCV10 in Nigeria.

## Electronic supplementary material

Table S1
