# Supplementary material for: Nasopharyngeal Pneumococcal Carriage in Nigeria: a two-site, population-based survey
Source: Sci Rep. 2018 Feb 22;8:3509. doi: 10.1038/s41598-018-21837-5 (PMC5823928; doi:10.1038/s41598-018-21837-5)
Supplement: Supplementary file 1 — Table S1 [file 41598_2018_21837_MOESM1_ESM.pdf]

Nasopharyngeal Pneumococcal Carriage in Nigeria: a two-site, population-based survey

Ifedayo M. O. Adetifa[1, 2, 3§], Aishatu L. Adamu[4], Angela Karani[2], Michael Waithaka[2], Kofo A. Odeyemi[5*], Christy A. N. Okoromah[3*], Mohammed M. Bello[4*], Isa S. Abubakar[4*], Victor Inem[5*], J. Anthony. G Scott[1,2]

[1]Department of Infectious Diseases Epidemiology, London School of Hygiene and Tropical Medicine, WC1E 7HT, London, UK

[2]Department of Epidemiology and Demography, KEMRI-Wellcome Trust Research Programme, Centre for Geographic Medicine-Coast, Kilifi, Kenya

[3] Department of Paediatrics and Child Health, College of Medicine University of Lagos/Lagos University Teaching Hospital, Idi-Araba, PMB 12003, Lagos, Nigeria

[4] Department of Community Medicine, Bayero University/Aminu Kano Teaching Hospital, PMB 3452, Kano, Nigeria

[5] Department of Community Medicine and Primary Care, College of Medicine University of Lagos/Lagos University Teaching Hospital, Idi-Araba, PMB 12003, Lagos, Nigeria

[§]Corresponding author

*These authors contributed equally

Table S1. Prevalent pneumococcal serotypes by location and age group

Kumbotso (rural)

Age group (years)	0-4		5-17		18-34		35-49		≥50	
	N	%	N	%	N	%	N	%	N	%
**Sample size**	295		288		145		76		66	
**Carriage**	271	91.9	228	79.2	77	53.1	34	44.7	33	50.0
**Serotype-specific prevalence***										
19F	48	17.7	16	7.0	7	9.1	1	2.9	1	3.0
6A	42	15.5	17	7.5	4	5.2	1	2.9	2	6.1
23F	31	11.4	15	6.6	2	2.6	0	0.0	1	3.0
6B	15	5.5	8	3.5	1	1.3	1	2.9	0	0.0
11A	10	3.7	7	3.1	5	6.5	0	0.0	1	3.0
14	9	3.3	5	2.6	1	1.3	5	14.7	0	0.0
15C	9	3.3	3	1.3	3	3.9	0	0.0	1	3.0
19A	8	3.0	3	1.3	0	0.0	0	0.0	0	0.0
4	8	3.0	6	2.6	3	3.9	0	0.0	0	0.0
23B	7	2.6	2	0.9	0	0.0	0	0.0	0	0.0
9V	7	2.6	10	4.4	3	3.9	0	0.0	0	0.0
21	6	2.2	6	2.6	2	2.6	1	2.9	4	12.1
34	6	2.2	9	3.9	1	1.3	2	5.9	6	18.2
NT	6	2.2	5	2.2	4	5.2	0	0.0	1	3.0
18C	5	1.8	5	2.2	2	2.6	0	0.0	0	0.0
Others	26	19.9	31	40.4	21	52.0	17	67.6	28	48.4

Pakoto (urban)

Age group (years)	0-4		5-17		18-34		35-49		≥50	
	N	%	N	%	N	%	N	%	N	%
**Sample size**	335		250		109		84		141	
**Carriage**	260	77.6	123	49.2	27	24.8	19	22.6	32	22.7
**Serotype-specific prevalence***										
19F	46	17.7	10	8.1	3	11.1	2	10.5	0	0.0
6B	35	13.5	11	8.9	2	7.4	1	5.3	0	0.0
6A	26	10.0	11	8.9	2	7.4	1	5.3	2	6.3
23F	25	9.6	14	11.4	2	7.4	0	0.0	3	9.4
19A	18	6.9	7	5.7	0	0.0	0	0.0	2	6.3
NT	15	5.8	8	6.5	1	3.7	2	10.5	2	6.3
14	12	4.6	3	2.4	1	3.7	2	10.5	2	6.3
15B	10	3.8	3	2.4	0	0.0	0	0.0	1	3.1
11A	9	3.5	9	7.3	2	7.4	1	5.3	1	3.1
3	8	3.1	3	2.4	1	3.7	1	5.3	6	18.8
35B	8	3.1	0	0.0	0	0.0	0	0.0	1	3.1
9V	5	1.9	4	3.3	0	0.0	0	0.0	0	0.0
11B	4	1.5	1	0.8	1	3.7	0	0.0	0	0.0
16F	4	1.5	2	1.6	0	0.0	3	15.8	2	6.3
21	4	1.5	0	0.0	0	0.0	0	0.0	1	3.1
Others	17	12.3	20	29.8	11	44.4	6	31.6	7	28.1
